# The Harveian Oration

**Published:** 1902-10-25

**Authors:** 


### The Hospital.

A Journal of

The Medical Sciences and Hospital Administration.

Vol. XXXIII.—No. 839.] Edited by Sir Henry Burdett, K.C.B., and Solomon C. Smith, M.D., M.R.C.P. [October 25, 1902

### THE HARVEIAN ORATION.

The Harveian Oration, delivered at the Royal College of Physicians last Saturday by Dr. Ferrier, was a masterly summary of all that has been made known, since the time of Harvey himself, with regard to the mechanism of those local variations of blood-supply which fulfil so many and such varied purposes in the animal economy. It was, of course, mainly a record of experimental research, somewhat scanty and barren in the earlier days, but becoming crowded, and sometimes even contradictory or confused, as our own period was approached and reached. The processes of excitation and of inhibition, the successive discoveries of the vaso-constrictor and vaso-dilator nerve filaments, and the evidences of the functions which they respectively discharge, were all graphically described, but the whole narrative was not ill calculated to recall the well-known words in which Newton likened himself to a child standing on the brink of a boundless ocean, and playing with a pebble which the waves had washed to his feet. As regards the nervous system and its functions, physiology has at yet done little more than map out the approaches to an unexplored country, in which, if we could enter it, we might expect to find the solution of many mysteries. The late Sir Benjamin Richardson was accustomed to maintain that the difference between courage and cowardice was mainly dependent upon the muscular strength of the left ventricle of the heart, and upon its power to maintain an adequate supply of blood to the brain in despite of the presence of some depressing agency. In certain circumstances, one kind of physical formation was more useful than another, and that was all. We question whether this hypothesis, at all events when stated thus baldly, could be maintained in the face of investigation; but there can be little doubt that strength or activity of heart would be at least one element in the production of the general result. The history related by Dr. Ferrier points, however, to the probability that the *vera causa* may lie farther back than the heart muscle as such, and that it may be dependent upon the character and the degree of the nerve stimulation which reaches its fibres, or the fibres of the muscles which control the amount of its blood-supply. And what then? When we have heaped Pelion upon Ossa, are we appreciably nearer to the secret into which we would penetrate?

The question which in this way may be reduced to its simplest terms is that which, perhaps of all others, is the most important to the progress and to the welfare of the human race. It underlies all conduct, all education, all moral praise or censure, much disease, and probably all, or nearly all, insanity. It underlies that great problem of free will or necessity which, from the very dawnings of philosophy, has perplexed the minds of men. To what extent do our decisions or our actions depend upon the temporary or the permanent character of the blood-supply of some nervous centre; and on what circumstances is this blood-supply itself dependent? Dr. Ferrier has so far dealt with the second question as to present us with a concise description of the ultimate mechanism concerned, but beyond this he cannot go. He tells us that it is not at present possible to explain the blush of shame or modesty. No one knows why, under such emotion, the arterioles of the cheek admit an increased quantity of blood. *A fortiori*, no one knows why the arterioles of some nerve centre should act in a similar manner, although it is impossible to doubt that they do, or that the functions of motion, sensation, and thought may in this way be modified. So far, therefore, physiology has only taken us one step in the direction of understanding conduct, or the intellectual conditions from which conduct springs. We now know, if not all, at least a good deal, about vaso-dilator and vaso-constrictor nerves; and we can see, so to speak, the hand by which vascular tension is controlled. We are not perceptibly nearer to the mind which is behind the hand, to the really effective cause of arterial contraction or dilatation, as these occur continually in our bodies. The experience recently gained with supra-renal extract seems to show that the determining influence may, after all, be chemical, the presence or absence, the excess or deficiency, of something prepared within the laboratory of our bodies. It is even conceivable that an analysis of the blood or tissues may some day throw light upon character or capacity, may explain, for example, that problem which was so well stated by the late Dr. Moxon, in his inquiry why a given man, not noticeably different in organisation from his fellows, became a sot. However this may be, it is at least probable, if not certain, that the discoveries of the future, with regard to human function, will be made either in the laboratory of the experimental physiologist or in that of the organic chemist; and it is these two classes of philosophers who must mainly bear the burden of Harvey's injunction to the Fellows of his College, that they should search out the secrets of Nature by way of experiment. We trust it is not impertinent to inquire to what extent the College has sought to qualify its Fellows for the complete fulfilment in action of a duty which has long been recognised in words? To put the question in another form: Is the education of the medical profession, which the College largely controls, such as adequately to prepare students for making discoveries themselves, or even for the most profitable application of the discoveries made by others?

